# Visualising and quantifying ‘excess deaths’ in Scotland compared with the rest of the UK and the rest of Western Europe

**DOI:** 10.1136/jech-2016-207379

**Published:** 2017-04-10

**Authors:** Jon Minton, Richard Shaw, Mark A Green, Laura Vanderbloemen, Frank Popham, Gerry McCartney

**Affiliations:** 1 College of Social Sciences, University of Glasgow, Glasgow, UK; 2 MRC/CSO Social and Public Health Sciences Unit, University of Glasgow, Glasgow, UK; 3 School of Environmental Sciences, University of Liverpool, Liverpool, UK; 4 Faculty of Medicine, Department of Primary Care and Public Health, Imperial College, London, UK; 5 NHS Health Scotland, Glasgow, UK

**Keywords:** DEMOGRAPHY, GEOGRAPHY, Health inequalities, Life course epidemiology, METHODOLOGY

## Abstract

**Background:**

Scotland has higher mortality rates than the rest of Western Europe (rWE), with more cardiovascular disease and cancer among older adults; and alcohol-related and drug-related deaths, suicide and violence among younger adults.

**Methods:**

We obtained sex, age-specific and year-specific all-cause mortality rates for Scotland and other populations, and explored differences in mortality both visually and numerically.

**Results:**

Scotland's age-specific mortality was higher than the rest of the UK (rUK) since 1950, and has increased. Between the 1950s and 2000s, ‘excess deaths’ by age 80 per 100 000 population associated with living in Scotland grew from 4341 to 7203 compared with rUK, and from 4132 to 8828 compared with rWE. UK-wide mortality risk compared with rWE also increased, from 240 ‘excess deaths’ in the 1950s to 2320 in the 2000s. Cohorts born in the 1940s and 1950s throughout the UK including Scotland had lower mortality risk than comparable rWE populations, especially for males. Mortality rates were higher in Scotland than rUK and rWE among younger adults from the 1990s onwards suggesting an age–period interaction.

**Conclusions:**

Worsening mortality among young adults in the past 30 years reversed a relative advantage evident for those born between 1950 and 1960. Compared with rWE, Scotland and rUK have followed similar trends but Scotland has started from a worse position and had worse working age–period effects in the 1990s and 2000s.

## Introduction

Successive generations living in Europe have tended to outlive the last,^1^ as mortality rates in infancy and at later ages fell.^2^ ^3^ In this marathon to greater longevity, Scotland is considered a laggard.^4^ By 2010, Scotland's life expectancy ranked lowest among countries in Western and Central Europe,^2^ with high rates of deaths due to substance abuse, suicide and violence among younger adults; and high rates of death due to cardiovascular disease, cancer and respiratory disease among older adults.^5–7^

Research has explored the role of four mortality phenomena—lagging life expectancy improvement, wide health inequalities, the rise of external causes of death, the causes of the excess mortality—to understand Scottish longevity trends.^8^ ^9^ This paper extends the research programme to consider the role of age, period and cohort (APC) effects in explaining Scotland's high mortality compared with its neighbours, and how this has changed from the period 1950 to 2010. Given Scotland is part of the UK, and so subject to similar social and economic conditions, and political and healthcare decisions and priorities as much of the rest of the UK (rUK), it is also important to compare the UK as a whole with the rest of Western Europe (rWE) to better understand whether APC and related effects observed in Scotland are also seen elsewhere in the UK. At the core of this paper, therefore, are three pairs of comparison: Scotland compared with rUK, Scotland compared with rWE and the UK compared with rWE.

These three pairs of comparison are made in two different ways: first visually using a form of heatmap we call a comparative level plot (CLP); and second numerically, by using the same age-specific mortality rate data to estimate the cumulative mortality in different years, for different birth cohorts, and by different ages, in each of the populations being compared.

## Methods

### Data

Death counts and population exposures (population sizes at risk of death) were extracted from the Human Mortality Database (HMD),^10^ by single year of age at death and calendar year, for the period 1950–2010 from birth to age 109, for Scotland, the rUK and for other European countries. These counts were arranged into a single rectangular data set using approaches detailed in a HMD technical report, and made use of country-specific files Deaths1_1.txt (1 year by 1 year Lexis squares) for death counts, and Exposures_1×1.txt files for population counts. For the birth cohort-based lifetable approximations detailed below, death count and exposure count data were extracted from earlier periods where available. Death counts and population exposures for the whole of the UK were produced by aggregating values for the same sex, year and age for England and Wales (HMD code GBRCENW), Scotland (GBR_SCO) and Northern Ireland (GBR_NIR). Western Europe, excluding the UK, comprised Austria (AUT), Belgium (BEL), Switzerland (CHE), East and West Germany (DEUTE and DEUTW), France (FRACNP), Republic of Ireland (IRL), Luxembourg (LUX) and the Netherlands (NLD). Data were available in the HMD for each of these countries for each year from 1950 to 2009, except for East and West Germany (first year 1956) and Luxembourg (first year 1960). Population and death counts for 2010 were not available for Austria, Ireland, Luxembourg and the Netherlands. Further details are provided in the web appendix.

### Measures calculated

Mortality rates at each age were calculated by dividing the aggregated death counts by aggregated population counts. For the CLPs, these were converted into log mortality rates using base 10, meaning that the values indicate the ‘number of zeroes’ in the mortality risk (ie, −3.0 means 1-in-1000, −2.0 means 1-in-100 and so on). What the CLPs show are differences in these log mortality rates between populations of the same age, sex and in the same year between the two populations being compared. This means that the mortality rate ratio for any value (v) on the surface can be calculated as 10^v^. So, for example, a value of 0.01 implies around a 2% increased risk (10^0.01^=1.023 to 3 decimal places), a value of 0.05 around a 12% increased risk (10^0.05^=1.122 to 3 decimal places), a value of 0.10 around a 26% increased risk and a value of 0.20 around a 60% increased risk. Lifetables for single or multiple country populations were calculated using the denominators in the Exposure_1×1.txt files within the HMD, and probabilities of death within 1 year age intervals calculated using equation 60 of the HMD methods guide, and using the recommended adjustments for mortality risk exposure in the first year of life.^11^ ^12^ Unadjusted estimates using values in the HMD Population.txt files as denominators were also calculated and available as an appendix. From these lifetables, cumulative probabilities of death by particular ages were calculated, and from this estimates of ‘excess deaths’ produced. One year by 1 year Lexis squares were used for both cohort-based and period-based estimates; for the cohort-based estimates, this was analogous to rearranging checkerboard squares so that diagonal lines become vertical, a simple illustration of which is provided in the web appendix. All analyses were performed using the R programming environment (Team RC. R: A language and environment for statistical computing. 2014).

### CLPs and Lexis surfaces

The CLPs have age across the vertical axis, year across the horizontal, and values—the differences in log_10_ mortality rates—for each of these age/year combinations as coloured cells. This particular arrangement of values is known as a Lexis surface,^13^ defined more formally as a Cartesian mapping of year, age and a third variable onto orthogonal axes.^14^ ^15^ Lexis surfaces provide a visual description of variation across (1) the magnitude of rates, (2) how rates vary by age, (3) temporal trends in rates, (4) the interaction between age and time (ie, cohort influences).^14^ ^16^ They form a useful first step in understanding the importance of APC influences and supplement more formal modelling approaches which are often troubled in separating out APC effects.^15^ ^17^ ^18^

A red–blue divergent colour scheme was used in the CLPs. For any two population mortality rates A and B, the shade shows the value d, defined as log_10_(B)**−**log_10_(A), equivalently log_10_(B/A), and is red if the value B is greater than the value A, and blue if A is greater than B. The shade indicates how big the differences are between B and A, with darker shades indicating greater differences and lighter indicating smaller differences; any ratio B/A will be as dark a shade of red as the ratio A/B will be blue. Neighbouring values on the Lexis surface were ‘smoothed’ slightly using a Gaussian filtering algorithm within the spatstat package in R to make patterns and trends in the data easier to identify.^19^ ^20^ CLPS using unsmoothed data are in the online appendix. The CLPs were produced using the Lattice and LatticeExtra packages.^21^ The same scale for year and age are used as this facilitates the identification of cohorts as they run at exactly 45° from the bottom left to top right in the figures. A total of 61 years are covered (1950–2010) and a maximum age of 90 is presented to avoid producing figures with a very high aspect ratio.

### Lifetable analysis

Approximate lifetables were produced for many cohorts, each of 100 000 people (50 000 males and 50 000 females) from birth to much older ages. Each year, some of this initial cohort ‘die’ according to age-specific mortality rates from the HMD, and the different population sizes remaining in place A (rUK or rWE) and place B (Scotland or UK) by particular ages are compared, producing estimates of cumulative ‘excess mortality’ in place B compared with place A by particular ages. The lifetables used to produce these excess mortality estimates were calculated both for periods (stationary cohorts), equivalent to vertical slices through the CLP, and for birth cohorts, equivalent to diagonal slices running at 45° through the CLP, based on aggregated death and population counts for full calendar years, for each period from 1950 to 2010, and for each birth year from 1930 to 1979. For brevity, the excess mortality estimates for each year of birth year are averaged over decades before being presented. The code used to produce these analyses are available in the web appendix.

## Results

### Comparative level plots

**Figure 1:**
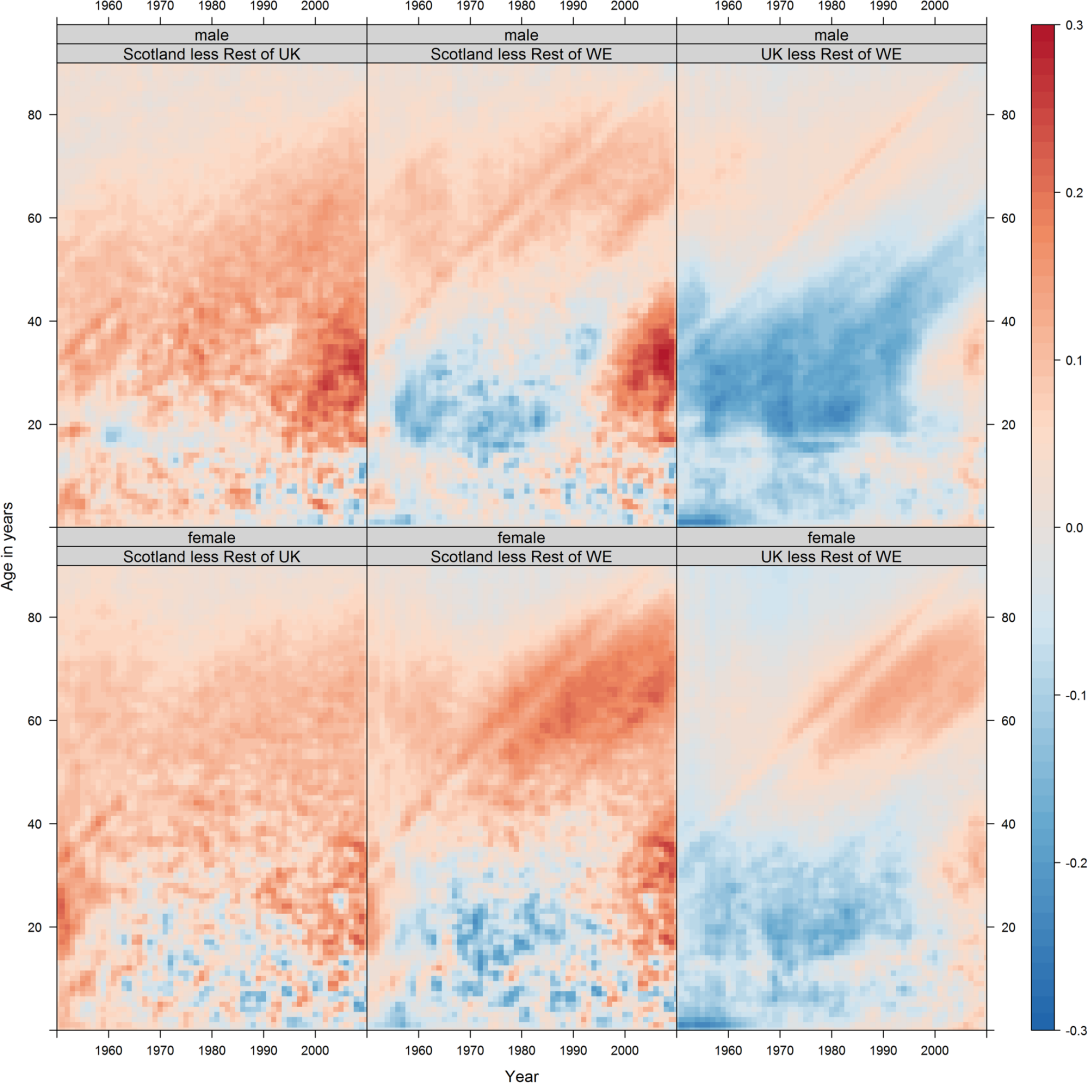
Historic differences between Scotland and rUK, the Golden Cohort, and young adults in the 1990s and 2000s. Note: long-term mortality differences between Scotland and rUK, all ages, 1950–1995. The overall colour within this region is pink/red, indicating slightly higher mortality risk in Scotland at almost all ages, throughout the period and for both sexes (figure 2, A0, A1, A2). Each of these differences at each age and in each year may be quite small, but they accumulate over the life course, leading to substantial differences in the life expectancy between Scotland and the other UK countries. The ‘Golden Cohort’, at all ages from early childhood to adulthood through to the start of retirement age, males in the UK born in the 1950s tended to experience lower mortality risk than age-matched Western European males. This difference is relatively consistent throughout but strongest (darkest blue) at two ages—young childhood and early adulthood (figure 2, B, B0). For *young adults in the 1990s and 2000s*, mortality risk in Scotland worsened relative to rUK, particular among males and from the mid-2000s onwards (figure 2, C, C*). The female/rUK difference over the same period/age range has not fallen as much further, suggesting this recent change is differentiated by sex. rUK, rest of the UK.

**Figure 2:**
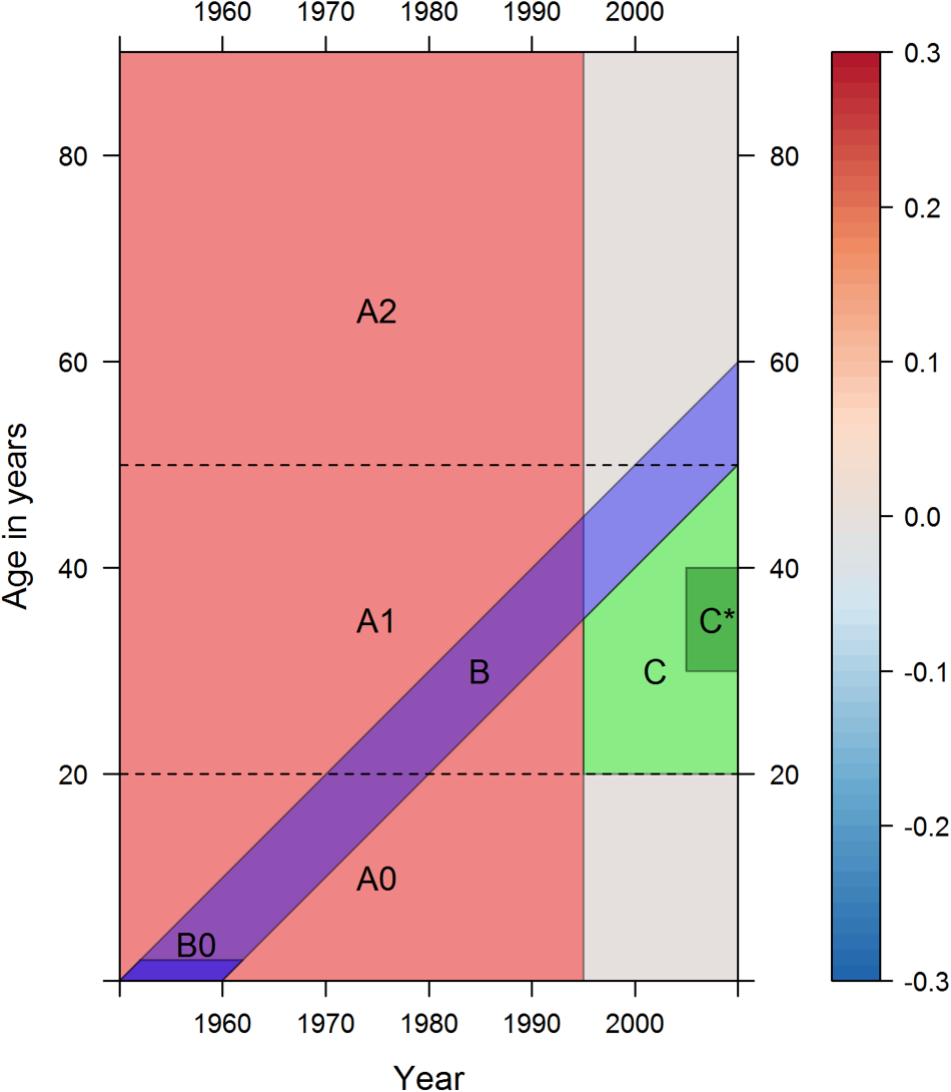
Three age/year regions of interest within figure 1. Region A is red, region B is blue and region C is green. WE, Western Europe.

Figure 1 shows a two row by three column series of CLPs. Each row is a different sex, and each column is a different comparison. In total, figure 1 represents more than 33 000 separate mortality risk differences, so to help navigate and discuss the figure we recommend considering each of the areas A, B and C, indicated in figure 2, in sequence.

Area A in figure 2 highlights the period 1950–1955. Over this period, the average colour in the Scot-rUK column is pink/red, suggesting Scots have had higher mortality risk at most ages in the latter half of the 20th century. Area A is partitioned into A0, covering childhood and adolescence; A1, covering early adulthood and A2, covering later adulthood. We can see from this that the difference tends to be highest in A1, early adulthood, for males, whereas for females Scot-rWE differences are greatest within A2, later adulthood. Area A in the UK-rWE column suggests similar mortality risk in childhood (A0), lower risk in early adulthood (A1), and similar (males) or higher (females) risk in later adulthood (A2).

Area B in figure 2 highlights cohorts born in the 1950. The subarea B0 highlights 0–3 years old, and the Scot-rWE and UK-rWE columns both show particularly low comparative mortality—a dark blue horizontal streak—at early ages. For UK-rWE males this comparatively low mortality appears to follow the cohort as they age, even as they enter their 60s. For UK-rWE females, and for Scot-rWE males and females, the 1950s cohort still appears distinct from neighbouring cohorts, but with effects that diminish with age and, for Scotland, to mitigate rather than prevent mortality risk disadvantage compared with rWE.

Area C in figure 2 highlights young adults, born after the 1950s, from 1995 onwards. The UK-rWE column shows that from the mid-1990s UK young adult mortality risk changed from lower (blue) to higher (red) than rWE, meaning a comparative advantage which had existed for nearly two generations was lost in recent years. Relative risk increased similarly for UK-rWE males and females, but slightly earlier and more severely in Scot-rWE, especially for males. Of particular concern are males aged between 30 and 40 years from 2005 onwards, marked as C*, which appears as a distinctly dark red section in figure 1, especially for males in the Scot-rUK and Scot-rWE, suggesting this recent deterioration is UK-wide but particularly acute in Scotland.

CLPs comparing Scotland against different European regions, rather than just Western Europe, shown in the web appendix, suggest important trends towards reduced older age mortality risks in Southern Europe, and troubling trends towards higher mortality risks, especially for males, in Eastern Europe. Additional shaded contour plots and CLPs are shown in the web appendix.

### Lifetable estimates

Table 1 shows the average number of expected cumulative excess deaths per 100 000 population by the ages indicated in the top row, for each of the decades from the 1950s to 2000s, equivalent to comparing vertical sections in the CLPs shown in figure 1. Table 2 shows the same, but for birth cohorts rather than years, and so is equivalent to comparing various diagonal sections both including and parallel to area B in figure 2 (the 1950s cohort).

**Table 1:** Period-based cumulative excess deaths per 100 000 population, by various ages

Decade	1	5	10	20	30	40	50	60	70	80
1950s	648	705	745	843	1077	1506	2303	3868	5240	4341
1960s	364	401	424	436	544	861	1669	3292	4932	4406
1970s	114	140	153	173	292	644	1520	3231	5040	4330
1980s	−17	−9	2	40	173	444	1223	3071	5481	5672
1990s	−16	−7	0	83	325	567	1226	2906	5560	6360
2000s	−35	−29	−24	80	367	807	1453	2795	5361	7203
*(A) Scotland—rUK*										
Decade	1	5	10	20	30	40	50	60	70	80
1950s	−312	−328	−314	−311	−312	−156	343	2069	4627	4132
1960s	75	72	65	−12	−162	−144	524	2409	4932	4368
1970s	111	110	97	−32	−160	−110	517	2703	5566	4820
1980s	40	35	33	3	−75	−72	359	2384	6473	7288
1990s	35	39	42	80	215	261	511	2043	5985	8792
2000s	46	51	56	152	435	910	1349	2327	5364	8828
*(B) Scotland—rWE*										
Decade	1	5	10	20	30	40	50	60	70	80
1950s	−888	−956	−977	−1063	−1273	−1505	−1724	−1407	−78	240
1960s	−250	−286	−314	−403	−652	−922	−987	−566	485	397
1970s	12	−13	−38	−184	−419	−688	−855	−211	1030	929
1980s	56	44	33	−32	−230	−475	−753	−396	1528	2183
1990s	50	46	42	6	−80	−257	−609	−600	954	3055
2000s	79	78	80	81	101	173	25	−218	502	2323
*(C) UK—rWE*										

These estimates are primarily for illustration as they are not based on birth cohorts, and are equivalent to taking decade-wide ‘vertical slices’ through data used to produce figure 1. The number in the top row is an age in years. The numbers in the columns below these ages show the number of cumulative excess deaths per 100 000 population (50 000 male and 50 000 female at birth) by that age, based on differences in age-specific/sex-specific mortality rates between (A) Scotland and rUK, (B) Scotland and rWE, (C) UK and rWE, in each of the 10 years within the decade indicated by the row. Positive values indicate excess deaths in Scotland (A, B) or the UK (C), and negative values indicate fewer deaths compared with rUK (A) or rWE (B, C).

rUK, rest of the UK; rWE, rest of Western Europe.

**Table 2:** Cohort-based cumulative excess deaths per 100 000 population, by various ages

Birth cohort	1	5	10	20	30	40	50	60	70
1930s	2274	2480	2551	2718	2809	3061	3764	5079	7000
1940S	1516	1647	1698	1708	1812	2093	2700	4039	
1950S	615	649	670	691	808	1010	1615		
1960S	363	401	419	456	627	982			
1970s	128	148	160	223	503				
*(A) Scotland—rUK*									
1930s	1593	1838	1845	1881	1737	1699	2100	3398	6143
1940s	−30	−51	−23	−92	−210	−174	113	1312	
1950s	−299	−333	−336	−454	−565	−600	−281		
1960s	77	80	68	9	30	354			
1970s	119	114	111	133	378				
*(B) Scotland—rWE*									
1930s	−377	−308	−363	−476	−703	−971	−1209	−1102	−91
1940s	−1366	−1504	−1522	−1600	−1814	−2035	−2302	−2323	
1950s	−846	−910	−932	−1068	−1286	−1506	−1738		
1960s	−247	−278	−306	−399	−534	−534			
1970s	7	−16	−30	−65	−76				
*(C) UK—rWE*									

The number in the top row is an age in years. The numbers in the columns below these ages show the number of cumulative excess deaths per 100 000 population (50 000 male and 50 000 female at birth) by that age, based on differences in age-specific/sex-specific mortality rates between (A) Scotland and rUK, (B) Scotland and rWE, (C) UK and rWE, for each birth cohort (by age of birth in single years) indicate by the birth cohort decade. Positive values indicate excess deaths in Scotland (A, B) or the UK (C), and negative values indicate fewer deaths compared with rUK (A) or rWE (B, C).

rUK, rest of the UK; rWE, rest of Western Europe.

Table 1A shows that mortality rates in Scotland were higher at all ages than in the rUK in the 1950s, with the excess larger with increasing age. However, over the subsequent decades, the excess mortality rates among those aged under 10 years reduced rapidly and consistently such that by the 1980s the rates were lower in Scotland. For those aged <20 years, the excess deaths declined to a low of 40 per 100 000 per year in the 1980s before subsequently increasing. A similar pattern was also seen for those aged <60 years where the excess declined from 1950 to 1980 before subsequently increasing again. When adults aged >60 years were included, there was decline in excess mortality in Scotland until the 1970s before it increased.

Table 2A shows that each Scottish birth cohort had higher mortality than the rUK throughout their life course, although earlier birth cohorts had consistently greater excesses than the later cohorts.

Table 1C shows that, compared with the rWE, the UK had lower mortality rates at all but the oldest ages in the 1950s, with substantially lower rates particularly evident among those aged <70 years. This relative advantage decreased over time, and decreased first at younger ages and then sequentially with increasingly older groups. The pattern is confirmed by the birth cohort analysis (table 2C), which shows that each cohort born from the 1940s onwards had a decreasingly small relative advantage over its life course. The exceptions to this pattern was a substantial excess mortality in the UK among those aged 70–80 years in the 1980s and a smaller relative advantage for the 1930s birth cohort in the UK than subsequent cohorts. Tables 1B and 2B, comparing Scotland with the rWE has some similarities to that of the UK. However, the relative advantages are generally lower, and disadvantages higher, throughout. Table 2A and B shows that the mortality rates for the Scottish birth cohort from the 1930s carries a particularly high risk throughout the life course in Scotland (in stark contrast to UK overall) and the 1940s birth cohort is only a little better; and mortality rates among those aged >40 years are consistently higher throughout the time series in Scotland. The birth cohort in Scotland born in the 1950s enjoys relatively lower mortality rates through the life course compared with both the UK (table 2B) and the rWE (table 2C).

## Discussion

### Main results

Mortality in Scotland has consistently been worse than rUK among adults over time, but the relative mortality advantage over the rWE has now been lost. The birth cohorts born in Scotland during the 1930s and 1940s had particularly high mortality rates over their life course relative to rUK and after the age of 50 years relative to rWE, in stark contrast to the relatively low mortality rates for those born in the 1950s in Scotland. Between the 1950s and the 1980s, the relative advantage in mortality rates enjoyed by Scotland and the UK compared with rWE declined, due to faster improvements among younger adults in rWE and the ageing of the 1950s UK birth cohort. During the 1990s and 2000s, the relative mortality rates among young adults became substantially worse, particularly among men. The Identification problem at the core of methodological debates in APC analysis means there will always be some ambiguity about the separate influence of APC effects on mortality and other epidemiological patterns.^18^ The log mortality divergence, particularly relating to regions C and C* in figure 2, could be thought of more as age–period interactions which emerged during the 1990s, more so than cohort effects related to differential experience at or near birth (in contrast to the patterns identified in regions B0 and B in figure 2). However, life course epidemiology emphasises that the concept of critical and sensitive periods, meaning stages in the life course which can have disproportionately large influence on later health, can cover ages later in childhood and adulthood, suggesting a broader ‘developmental origins of health and disease’ perspective should be considered in addition to a narrower ‘foetal origins of health and disease’ perspective focused around the gestational period.^22^ In particular, the fact that divergence in comparative mortality between Scotland and rUK, and rWE, tends to begin after the age of 18 suggests that cohorts can also be defined by differential experiences on the ‘onset of adulthood’, and that the transition from childhood to adulthood should also be considered a critical period from this perspective. Divergence between sex-specific and deprivation-specific risk of suicide in Scotland, for example, also tends to begin at the transition into adulthood, in particular among cohorts first exposed to the UK's neoliberal labour market reforms of the 1980s and 1990s when reaching adulthood, again suggesting this age as a critical stage in the life course.^23^

The methods employed here have identified birth cohorts in Scotland and the UK with vastly different mortality experiences over their life course, and complex patterns of divergence since the 1990s, which are not easily identified from mortality summary statistics such as life expectancy or age-standardised mortality rates, thereby highlighting their utility.

### Strengths and weaknesses

This is the first study to visualise age–year mortality data for Scotland in comparison to populations beyond the UK. These data ‘maps’ reduce the need to use summary statistics which can hide important APC effects,^24^ and are efficient and effective at presenting many separate values. CLPs used allow thousands of data points to be visualised at the same time, reducing the need to aggregate data into coarser age categories, cohorts and periods. Aggregating mortality rates may lead to misleading conclusions which the Lexis surface approaches used here would have avoided. Our CLPs show differences in log mortality rather than differences in mortality, effectively relative differences (B/A) rather than absolute differences (B**−**A); if absolute differences were presented then the rise in young adult mortality (figure 2 features C and C*) would have been less apparent. CLPs showing absolute differences are presented in the web appendix.

The source of data is robust and the risk of error or systematic bias is low (although, as with all mortality data, there are some potential difficulties in interpreting mortality rates during wartime with the exclusion of some deaths in conflict).^12^ The R code used to produce these analyses are made freely available to other researchers.

The lifetables following particular birth cohorts through time use period-based Lexis squares, rather than being composed of Lexis triangles which allow true population and death counts for specific birth cohorts to be estimated more precisely, although in practice the effect of using Lexis squares for estimating cohorts tends to be very small.^25^ Further research should investigate the effect that using Lexis squares rather than Lexis triangles has on these estimates.

The data have been smoothed slightly using an image processing algorithm before being plotted, and the appearance of the CLP necessarily depends partly on the degree and type of smoothing applied. Further research could investigate the influence the type and level of smoothing using both the spatstat package used here, the alternative MortalitySmooth package, and other approaches detailed elsewhere.^19^ ^20^ ^26^ Mortality data are a relatively insensitive measure of health status, particularly at younger ages, and quite large differences in mortality rates are obscured by the relatively large bands between colours on the charts.

The methods used and developed here can be used to explore differences in cause-specific mortality over time, at different ages, and between populations both within and between countries, and we encourage their use in this way, as well as for comparing both all-cause and cause-specific mortality trends in different countries and regions.

### Comparison with other studies

Analyses of demographic and epidemiological data focused on England and Wales, and the UK overall, have identified cohorts born between around 1925 and 1945 as having experienced greater mortality rate improvements than earlier or later cohorts.^27^ ^28^

This paper confirms the findings of others in terms of the emergence of cohorts with higher mortality in Scotland from the 1980s,^2^ ^6^ ^29^ ^30^ the intermediate position of Scotland between Eastern and Western Europe,^2^ ^4^ and the relatively low mortality in childhood in Scotland as in most other affluent world nations.^5^ The long-term impact (birth cohort effect) of the 1918 influenza epidemic has been noted in Europe^31^ and elsewhere,^32^ ^33^ but the positive generational birth cohort effect associated with the ‘baby boomers’ (born 1950–1960) in Scotland is new. Mackenbach's^34^ recent work exploring the convergence and then divergence in mortality experiences across Europe using summary statistics similarly identified the increased mortality among young adults in Eastern Europe and Scotland; the visual method employed here adds APC interactions. A recent paper by Seaman *et al*^35^ has addressed similar issues to this paper by comparing the changing relationship between period life expectancy and lifespan variation in Scotland compared with England and Wales.

The relative rise in mortality in young adults in Scotland is known to be related to increases in alcohol-related and drug-related deaths, suicide and violence, as well as wide inequalities across socioeconomic groups, and at older ages due to higher rates of cardiovascular, cancer and respiratory mortality.^4^ ^36^ The causes of these mortality patterns have been discussed at length elsewhere.^8^ ^29^

### Implications

Mortality trends in Scotland both followed and lagged those in the rUK, which worsened compared with rWE. Understanding why this relative decline occurred is important for improving health in Scotland and the rUK.^9^ The high relative mortality seen in young adults in the 1990s and 2000s in Scotland and elsewhere in the UK should be a source of concern, especially if this elevated mortality has a cohort component to it, and elevated relative mortality risk is ‘carried’ to older ages where absolute mortality risk is much higher. These methods could also be applied to understanding different populations within the same country or region, such as more and less socioeconomically deprived populations, and for exploring differing trends in cause-specific mortalities.

**What is already known on this subject**

- Substantively, it is known that mortality rates at many ages are high in Scotland compared with similarly developed countries, and have been for many decades. Methodologically, it is known that summary statistics such as period life expectancies can hide information about age-specific and year-specific mortality risks, which may indicate the presence of particular types of mortality risk; it is also known that heat maps and related approaches can be used to visually represent large numbers of age–year group-specific mortality risks, and so can help to reveal whether age, period or cohort effects are responsible for Scotland's poor overall mortality.

**What this study adds**

- Comparative level plots allow thousands of mortality risk comparisons to be made, and synthetic cohort approaches quantify the cumulative impact of differences in mortality risk. Period-based estimates suggest excess mortality by age 50 in Scotland compared with the rest of the UK (rUK) reduced from the 1950s to 2000s, but rose by age 80 over the same period, highlighting Scottish older age excess mortality. Compared with the rest of Western Europe cumulative mortality by age 80, in Scotland and the UK overall, rose from the 1950s to 2000s but is more than three times as high in Scotland than the rUK. Cumulative mortality by age 50 was historically lower in Scotland and the UK overall compared with the rest of Western Europe but became higher in Scotland in the 1990s and higher in the UK in the 2000s.
